# miR-155-5p Promotes Oxalate- and Calcium-Induced Kidney Oxidative Stress Injury by Suppressing MGP Expression

**DOI:** 10.1155/2020/5863617

**Published:** 2020-03-04

**Authors:** Kehua Jiang, Jianxin Hu, Guangheng Luo, Dalong Song, Peng Zhang, Jianguo Zhu, Fa Sun

**Affiliations:** ^1^Department of Urology, Guizhou Provincial People's Hospital, Guiyang, China; ^2^Guizhou University, Guiyang, China; ^3^Department of Urology, Panzhou People's Hospital, Panzhou, Guizhou, China

## Abstract

Oxalate and calcium are the major risk factors for calcium oxalate (CaOx) stone formation. However, the exact mechanism remains unclear. This study was designed to confirm the potential function of miR-155-5p in the formation of CaOx induced by oxalate and calcium oxalate monohydrate (COM). The HK-2 cells were treated by the different concentrations of oxalate and COM for 48 h. We found that oxalate and COM treatment significantly increased ROS generation, LDH release, cellular MDA levels, and H_2_O_2_ concentration in HK-2 cells. The results of qRT-PCR and western blot showed that expression of NOX2 was upregulated, while that of SOD-2 was downregulated following the treatment with oxalate and COM in HK-2 cells. Moreover, the results of miRNA microarray analysis showed that miR-155-5p was significantly upregulated after oxalate and COM treated in HK-2 cells, but miR-155-5p inhibitor treatment significantly decreased ROS generation, LDH release, cellular MDA levels, and H_2_O_2_ concentration in HK-2 cells incubated with oxalate and COM. miR-155-5p negatively regulated the expression level of MGP via directly targeting its 3′-UTR, verified by the Dual-Luciferase Reporter System. In vivo, polarized light optical microphotography showed that CaOx crystal significantly increased in the high-dose oxalate and Ca^2+^ groups compared to the control group. Furthermore, IHC analyses showed strong positive staining intensity for the NOX-2 protein in the high-dose oxalate and Ca^2+^-treated mouse kidneys, and miR-155-5p overexpression can further enhance its expression. However, the expression of SOD-2 protein was weakly stained. In conclusion, our study indicates that miR-155-5p promotes oxalate- and COM-induced kidney oxidative stress injury by suppressing MGP expression.

## 1. Introduction

Urolithiasis is a worldwide disease with calcium oxalate as the main component, along with ever-increasing morbidity [1, 2]. Calcium oxalate (CaOx), which is the main component of nephrolithiasis, can lead to increased intrarenal inflammation and kidney tubular cell injury and consequentially induce more CaOx crystal deposition, which is associated with oxidative stress injury and reactive oxygen species (ROS) [3]. Many recent studies have demonstrated that excessive oxalate or CaOx crystals in urine could result in the oxidative stress injury of renal tubular epithelial cells, and that the enormous amount of important free radicals, mainly ROS was induced by the response of renal tubular epithelial cells to the injury, which contributed importantly to the formation of CaOx stone [4–7]. Inhibition of renal inflammation response and oxidative stress has been identified as a potential strategy for the treatment of CaOx.

Previous studies indicated that oxidative stress injury plays an indispensable role in urolithiasis [8, 9]. Although the underlying mechanism is not clear, many studies have found that microRNAs (miRNAs) are closely related to oxidant stress injury as well as the pathogenesis of kidney stones; besides, they are promising and potential therapeutic targets or biomarkers for CaOx. Moreover, many studies showed that miRNAs could inhibit cell crystal adhesion or deposition in vitro and in vivo, such as miR-34a, miR-20b, and miR-30c [10, 11]. Our previous study also found that the interaction between H19 and miR-216b promotes calcium oxalate nephrocalcinosis-induced renal tubular epithelial cell injury and oxidative stress injury via HMGB1/TLR4/NF-*κ*B pathway [12]. Another study demonstrated that miR-23 decreased calcium oxalate crystal deposition and injury in the kidney [13]. A recent study demonstrated that nine miRNAs were differentially expressed in urine of CaOx patients compared with normal population [14]. It can be seen that miRNAs play a variety of roles in CaOx stone formation.

In our study, we identified 13 miRNAs after HK-2 cells were treated by oxalate and COM by a microarray technique, and miR-155-5p was significantly upregulated in two groups. Recently, miR-155-5p has been demonstrated that can induce ROS generation through downregulation of antioxidation-related genes in mesenchymal stem cells [15]. In another study, the results showed that miR-155-5p inhibit the IL-13-induced proliferation and migration of human bronchial smooth muscle cells [16]. In addition, miR-155 could aggravate the impaired autophagy of pancreatic acinar cells through targeting Rictor [17]. Taken together, these findings reveal that miR-155-5p functions in a cell type-specific manner like other miRNAs. However, its functions in oxalate- and CaOx crystal-induced renal tubular epithelial cells injury remain unknown. Therefore, in this study, our purpose is to elucidate the effect of miR-155-5p in the pathogenesis of CaOx induced by oxalate and CaOx crystals, and to clarify the underlying mechanism.

## 2. Materials and Methods

### 2.1. Cell Culture

Normal primary renal tubular HK-2 cell line was bought from China Center for Type Culture Collection (Wuhan, China). HK-2 cells were cultured in RPMI 1640 medium (Gibco, USA) with 10% fetal calf serum (FCS, Gibco) in an atmosphere of 5% CO_2_ at 37°C in cell humidified incubator. In this study, we followed the methods of Gao et al. [18], Liu et al. [12], and Song et al. [19]; when the cell confluence reached 65%, oxalate (0.2 *μ*mol/L, 0.6 *μ*mol/L) and COM (100 *μ*g/mL, 500 *μ*g/mL) crystals were added into HK-2 cells for study.

### 2.2. Cell Transfection

miR-155-5p mimics and inhibitor were bought from RiboBio (Guangzhou, China). MGP siRNAs and nontargeting siRNA were purchased from Gene Pharma (Shanghai, China). According to the manufacturer's protocol, 50 nM of miR-155-5p mimics and 100 nM of miR-155-5p inhibitor were transfected into the HK-2 cells by Lipofectamine 3000 (Invitrogen, China) in a 6-well cell plated. 100 nM of MGP siRNA was used to knockdown endogenous MGP following the manufacturer's instructions.

### 2.3. Real-Time Quantitative RT-PCR (qRT-PCR)

We followed the methods of Liu et al. [12]. The total RNA was extracted by TRIzol reagents (Invitrogen, USA) following the manufacturer's instructions. cDNAs were compounded by the Prime Script RT reagent kit (Takara). Then, qPCRs were conducted using 7500 Real-Time PCR System (Applied Biosystems) with SYBR Green PCR Master Mix (Applied Biosystems, USA) for mRNA analysis, and the expressions of MGP, NOX-2, and SOD-2 were normalized by GAPDH. qPCRs were conducted using the TaqMan microRNA Reverse Transcription kit (Applied Biosystems, USA) following the manufacturer's instructions for miRNA analysis; the expression of miR-155-5p was normalized by U6. The primer sequences are shown below:

miR-155-5p (F: 5′-GGTGCATTGTAGTTGCATTGC-3′, R: 5′-GTGCAGGGTCCG AGGTATTC-3′); U6 (5′-GCGCGTCGTGAAGCGTTC-3′, R: 5′-GTGCAGGGTCCG AGGT-3′); MGP (F: 5′-CTCAGTGTCAACATCTGACAG-3′, R: 5′-CATATGCTG CTCCTGCTGATC-3′); NOX2 (F: 5′-GTCACACCCTTCGCATCCATTCTCA AGTCAGT-3′, R: 5′-CTGAGACTCATCCCAGCCAGTGAGGTAG-3′); SOD-2 (F: 5′-GCTCCGGTTTTGGGGTATCTG-3′, R: 5′-GCGTTGATGTGAGGTTCCAG-3′); and GAPDH (F: 5′-ATGGGGAAGGTGAAGGTCG-3′, R: 5′-GGGGTCATTGATGGCA ACAATA-3′).

### 2.4. Western Blotting

Cell lysis and total protein extraction by RIPA buffer in 6-well cell culture plate. The protein concentration each sample was measured using the Bradford assay (Bio-Rad, USA). And then, the separation of protein is accomplished with 10% SDS-PAGE gel and electro-transferred to ECL nitrocellulose membranes; BSA with 0.1% Tris-buffered saline-Tween 20 (TBST) was applied to block the membranes for 2 h. The membranes of each sample were washed with TBST for 5 min and incubated overnight with MGP primary antibody (1 : 2000 dilution) and GAPDH primary antibody (1 : 3000 dilution). The membranes were subsequently incubated with matched secondary antibodies (1 : 3000 dilution) for 2 h at room temperate after they were washed with TBST three times. In the end, the ECL western blot analysis substrate was used to quantify the results.

### 2.5. Luciferase Reporter Assay

TargetScan and miRwalk were used for the bioinformatics algorithms. Wild-type 3′-UTR of MGP and mutant controls were constructed and inserted into the psiCheck2 Luciferase vector (Promega, USA). And then, the MGP-mutant or MGP-wild-type and miR-155-5p mimics were co-transduced into HK-2 cells by Lipofectamine 3000. Luciferase activity was detected using a Dual-Luciferase Reporter Assay System (Promega Corporation Madison, USA) based on the manufacturer's protocol after 48 h.

### 2.6. Experimental Mouse Model

We followed the methods of Liu et al. [12]. All animal experiments were performed according to protocols, approved by the Ethical Committee of Guizhou Provincial People's Hospital, and followed the rules of the National Institutes of Health Guide for the Care and Use of Laboratory Animals. C57BL/6 male mice (14 weeks old) were bought from the experimental animal center of Tongji Hospital (Wuhan, China) and used in the experiments. C57BL/6 male mouse was induced by intraperitoneal vehicle (saline) or glyoxylate (glyoxylic acid (GA)) (75 mg/kg) every day for two weeks to establish the CaOx crystal model. In the miR-155-5p, intervention groups and mice were injected with miR-155-5p agonist or antagonist through the tail vein. Eight weeks after the treatment, the mice were sacrificed and used in the study.

### 2.7. Polarized Light Optical Microphotograph for Kidney CaOx Crystals

We followed the methods of Liu et al. [12]. CaOx crystal deposition in the kidneys was determined using polarized light optical microphotography (Zeiss, Oberkochen, Germany). The percentage of crystal deposition area of each kidney section was quantified using Image-Pro Plus (Media Cybernetics, Inc., Bethesda, MD).

### 2.8. Measurement of Intracellular ROS Using Flow Cytometry

We followed the methods of Liu et al. [12]. Cells were cultured in a 6-well plate and treated by oxalate and COM as mentioned before. Cells were harvested and treated with DCFH-DA for 0.5 h, and then washed with PBS, and the intracellular ROS production was measured by flow cytometry with excitation and emission at 485 and 538 nm, respectively (Mod Fit software).

### 2.9. Lactate Dehydrogenase (LDH), Malondialdehyde (MDA), H_2_O_2_, and Superoxide Dismutase (SOD) Level Determination

We followed the methods of Liu et al. [12]. The HK-2 cells were harvested, and then the centrifugation of the released levels of LDH in the supernatants was measured using an LDH Assay Kit (Beyotime Biotech, China). MDA was measured using an MDA Kit (Beyotime Biotech, China). The H_2_O_2_ concentrations were measured using chemiluminescence methods (Beyotime Biotech, China), and the results were detected at 595 nm.

### 2.10. Statistical Analysis

All data were presented as mean values ± SD (standard deviations). One-way ANOVA was utilized to analyze the results between treated and control groups carried out with GraphPad Prism 5.0. The value of *P* < 0.05 was considered to have statistical significance.

## 3. Results

### 3.1. Oxalate Crystal- and COM Crystal-Induced Renal Tubular Epithelial Cell Oxidative Stress Injury in HK-2 Cells

**Figure 1:**
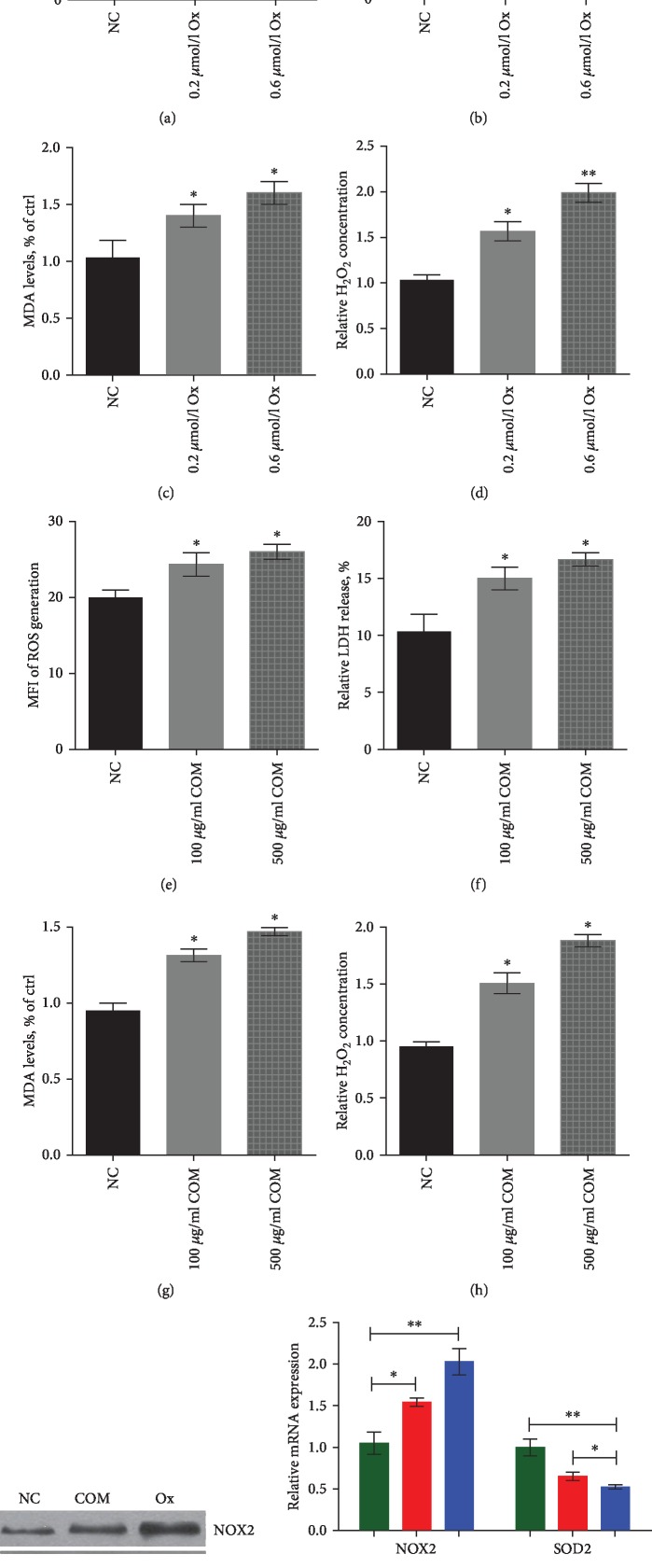
Oxalate crystal- and COM crystal-induced renal tubular epithelial cell oxidative stress injury in HK-2 cells. (a–d) Oxalate treatment significantly increased ROS generation, LDH release, cellular MDA levels, and H_2_O_2_ concentration in HK-2 cells. (e–h) COM crystal treatment significantly increased ROS generation, LDH release, cellular MDA levels, and H_2_O_2_ concentration in HK-2 cells. (i, j) Western blot and qRT-PCR were used to detect the expression of NOX2 and SOD-2 following the treatment with oxalate and COM in HK-2 cells. ^∗^*P* < 0.05 and ^∗∗^*P* < 0.01 vs. NC group.

To investigate oxalate and COM effects on the kidney cell injury, the HK-2 cells were treated by the different concentrations of oxalate and COM for 48 h. We found that oxalate and COM treatment significantly increased ROS generation, LDH release, cellular MDA levels, and H_2_O_2_ concentrations in HK-2 cells (Figures 1(a)–1(h)).

Using western blot and qRT-PCR analyses, the expression of NOX2 was shown to be upregulated, while that of SOD2 was downregulated following the treatment with oxalate and COM in HK-2 cells (Figures 1(i) and 1(j)).

### 3.2. miR-155-5p Promotes Oxalate- and COM-Induced Oxidative Stress Injury in HK-2 Cells

**Figure 2:**
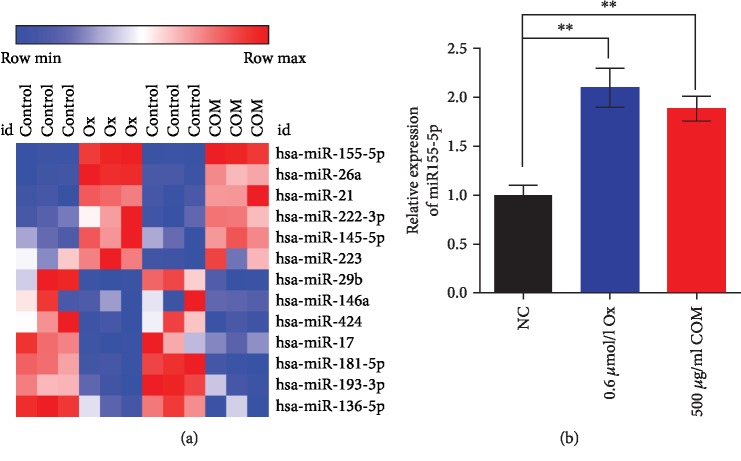
miR-155-5p significantly upregulated after oxalate and COM treated in HK-2 cells. (a) miRNA microarray analysis was applied to determine the expression level of miRNAs after oxalate and COM treated in HK-2 cells. (b) qRT-PCR analysis was performed to detect the expression of miR-155-5p following the treatment with oxalate and COM in HK-2 cells. ^∗∗^*P* < 0.01 vs. NC group.

To determine whether oxalate and COM affect the expression levels of miRNAs in HK-2 cells, we determined miRNA levels in HK-2 cells treated with 0.6 *μ*mol/L oxalate and 500 *μ*g/mL COM. The results of miRNA microarray analysis showed that 13 miRNAs could only be identified after HK-2 cells were treated by oxalate and COM, and miR-155-5p was significantly upregulated in two groups; we, therefore, choose miR-155-5p as a miRNA of interest for study (Figures 2(a) and 2(b)).

**Figure 3:**
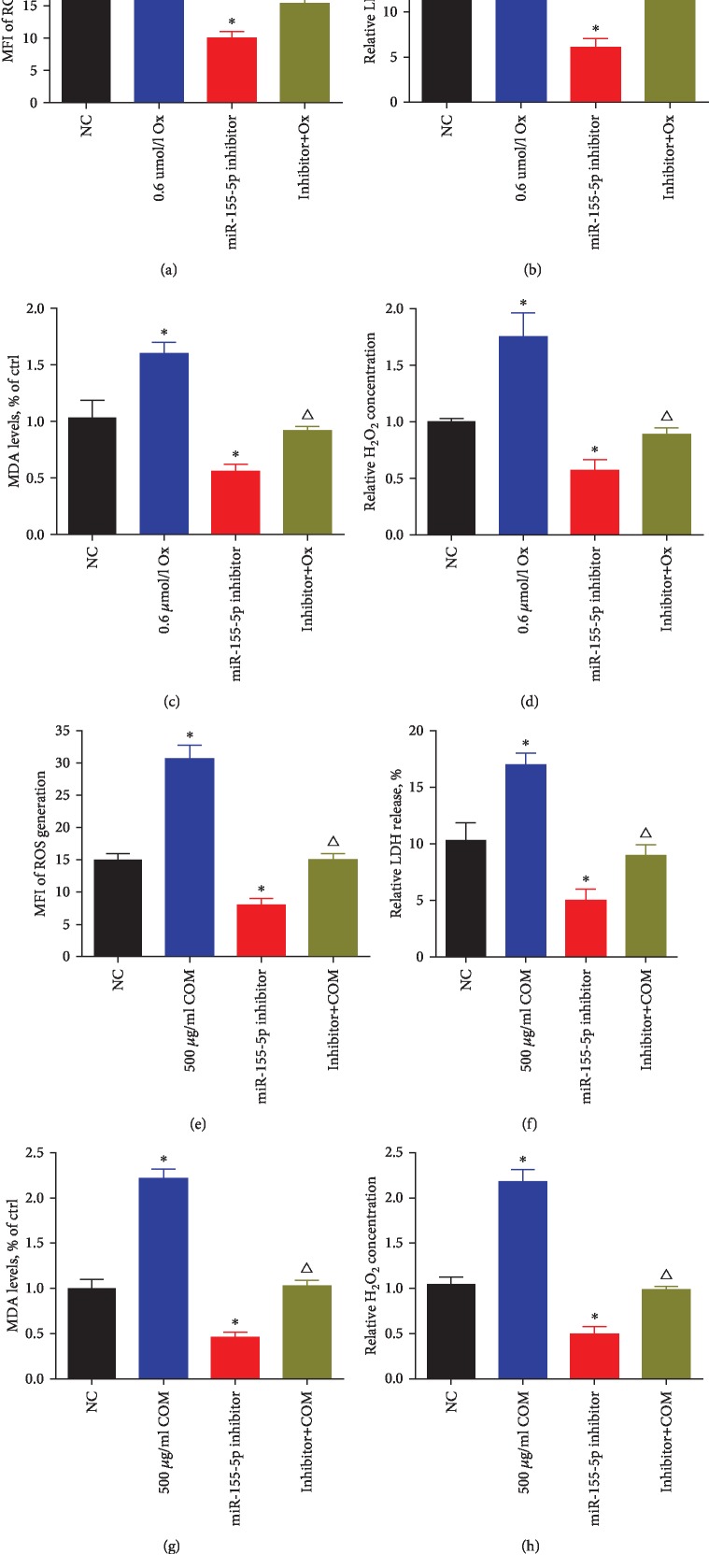
miR-155-5p promotes oxalate- and COM-induced oxidative stress injury in HK-2 cells. (a–d) miR-155-5p inhibitor treatment significantly decreased ROS generation, LDH release, cellular MDA levels, and H_2_O_2_ concentration in HK-2 cells incubated with oxalate. (e–h) miR-155-5p inhibitor treatment significantly decreased ROS generation, LDH release, cellular MDA levels, and H_2_O_2_ concentration in HK-2 cells incubated with COM. ^∗^*P* < 0.05 and ^∗∗^*P* < 0.01 vs. NC group; ^△^*P* < 0.05 vs. oxalate or COM group and miR-155-5p inhibitor group.

**Figure 4:**
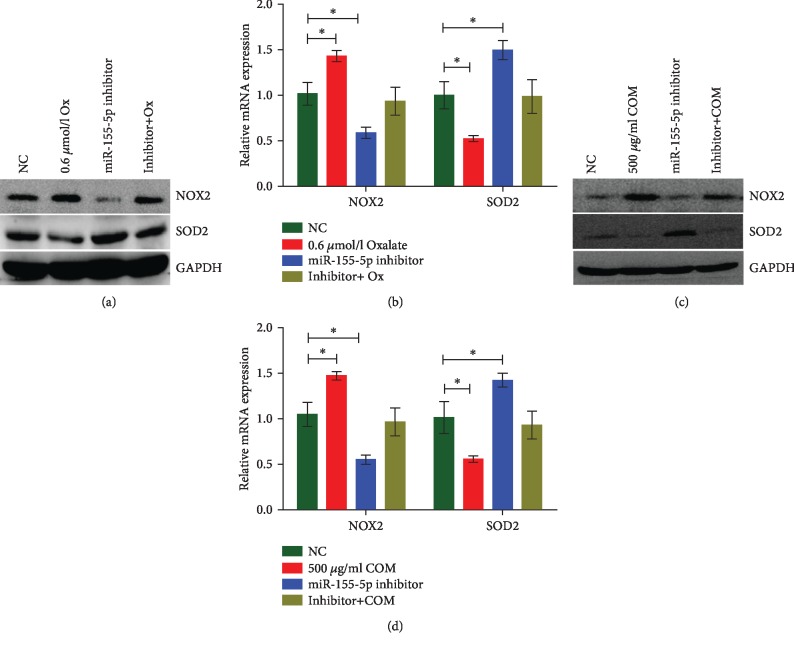
miR-155-5p promotes oxalate- and COM-induced oxidative stress injury in vitro. (a, b) Western blot and qRT-PCR analyses were performed to detect the expression of NOX-2 and SOD-2 following the treatment with oxalate and miR-155-5p inhibitor in HK-2 cells. (c, d) Western blot and qRT-PCR analyses were performed to detect the expression of NOX-2 and SOD-2 following the treatment with COM and miR-155-5p inhibitor in HK-2 cells. ^∗^*P* < 0.05 and ^∗∗^*P* < 0.01 vs. NC group.

From the investigation of miR-155-5p effects on the oxalate- and COM-induced kidney cell injury, miR-155-5p inhibitor treatment significantly decreased ROS generation, LDH release, cellular MDA levels, and H_2_O_2_ concentration in HK-2 cells incubated with oxalate and COM (Figures 3(a)–3(h)). Using western blot and qRT-PCR analyses, the expression of NOX2 was shown to be downregulated, while that of SOD-2 was upregulated following the treatment with miR-155-5p inhibitor in HK-2 cells, but this effect can be reversed by oxalate or COM (Figures 4(a)–4(d)).

### 3.3. MGP Is a Direct Target of miR-155-5p in HK-2 Cells

**Figure 5:**
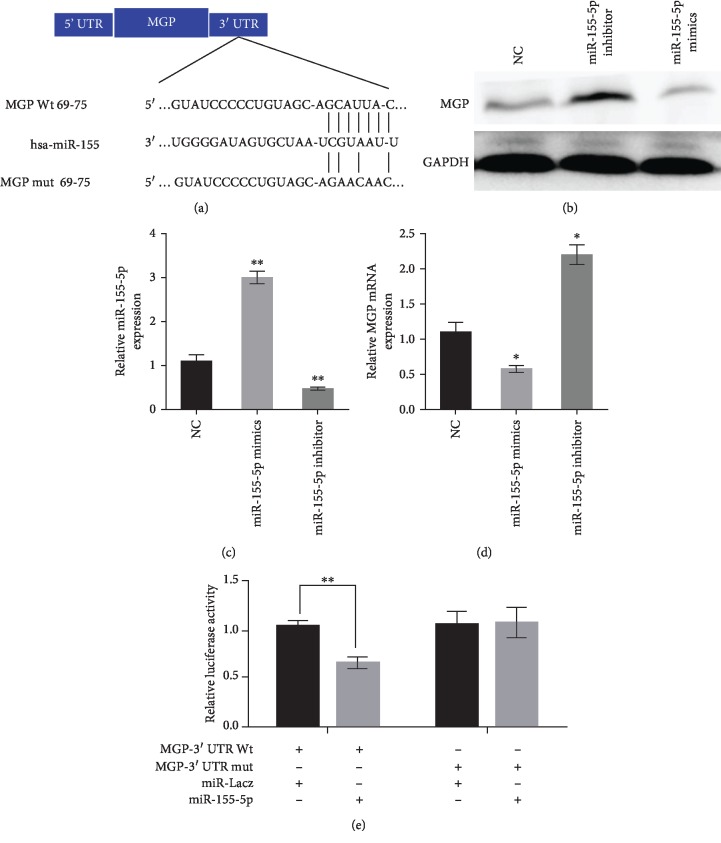
MGP is a direct target of miR-155-5p in HK-2 cells. (a) Schematic diagram of the mutant and wild-type seed sequences of miR-155-5p targeting the 3′-UTR of MGP. (b, d) Western blot and qRT-PCR analyses were performed to detect the protein expression of MGP after miR-155-5p inhibitor and mimics treatment in HK-2 cells. (c) qRT-PCR analysis was performed to detect the expression level of miR-155-5p in HK-2 cells. (e) Luciferase reporters harbouring putative target sites in the WT and mutant 3′-UTR of MGP were cotransfected with miR-155-5p mimics or inhibitor in HK-2 cells. ^∗^*P* < 0.05 and ^∗∗^*P* < 0.01 vs. NC group.

miRanda, miRBase, and TargetScan were applied to predict the potential targets of miR-155-5p. The results of bioinformatics algorithms indicated that MGP was the potential direct target of miR-155-5p (Figure 5(a)). The Dual-Luciferase Reporter System showed that miR-155-5p mimics significantly reduced the luciferase activity of MGP-WT, while miR-155-5p failed to repress the expression of luciferase containing a mutant binding site (Figures 5(b)–5(e)). The results of this experiment show that miR-155-5p negatively regulated the expression of MGP via directly targeting its 3′-UTR.

### 3.4. MGP Reversed the Effect of miR-155-5p on Oxalate- and COM-Induced Renal Tubular Epithelial Cell Injury

**Figure 6:**
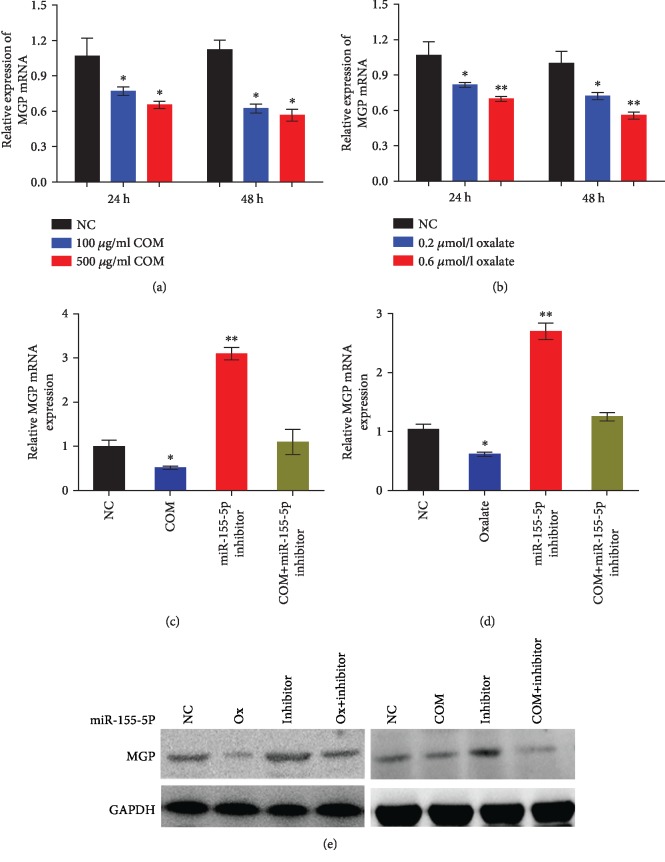
MGP reversed the effect of miR-155-5p on oxalate- and COM-induced renal tubular epithelial cell injury. (a, b) qRT-PCR analysis was performed to detect the expression level of MGP following oxalate and COM treatment in HK-2 cells. (c–e) qRT-PCR and western blot analyses were performed to detect the expression level of MGP after miR-155-5p inhibitor and oxalate or COM treatment in HK-2 cells. ^∗^*P* < 0.05 and ^∗∗^*P* < 0.01 vs. NC group.

Furthermore, qRT-PCR analyses of oxalate- and COM-treated HK-2 cells showed a downregulation of MGP expression in a time-dependent manner (Figures 6(a) and 6(b)). Moreover, qRT-PCR and WB results showed that high levels of oxalate or COM resulted in decreased expression levels of MGP protein in HK-2 cells, but this effect can be reversed by miR-155-5p inhibitor (Figures 6(c)–6(e)).

### 3.5. miR-155-5p Promotes Oxalate- and Ca^2+^-Induced Oxidative Stress Injury In Vivo

**Figure 7:**
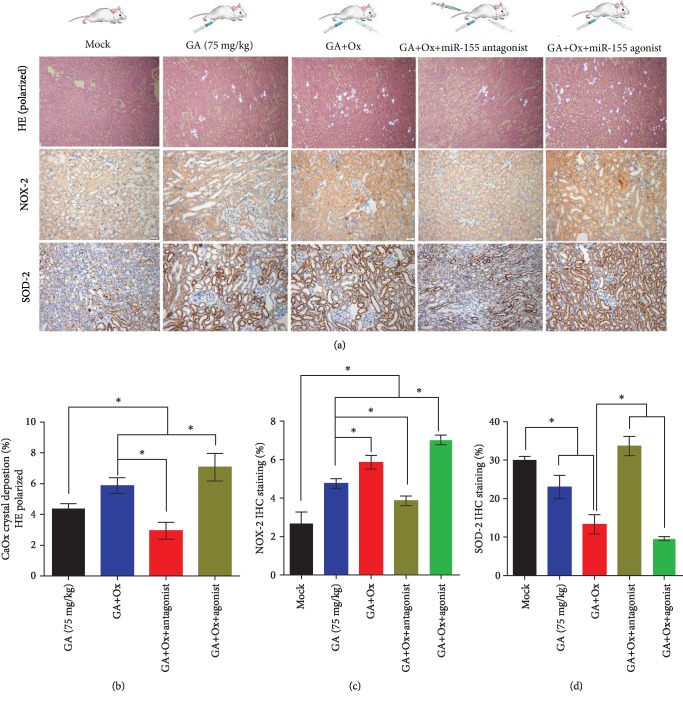
miR-155-5p promotes oxalate-induced oxidative stress injury in vivo. (a) CaOx deposition in the corticomedullary junction area was measured by hematoxylin and eosin (HE) staining (polarized light microscopy), and immunohistochemical (IHC) analysis of kidney NOX2 and SOD-2 expression was performed in oxalate combined with miR-155-5p agonist- or antagonist-injected CaOx crystal mouse model (magnification, ×200; scale bar, 50 *μ*m). (b–d) Quantification of CaOx crystal deposition level in kidney and quantification of IHC staining of NOX2 and SOD-2 proteins after oxalate, miR-155-5p agonist, antagonist, or the combination of these treatments. ^∗^*P* < 0.05 and ^∗∗^*P* < 0.01.

**Figure 8:**
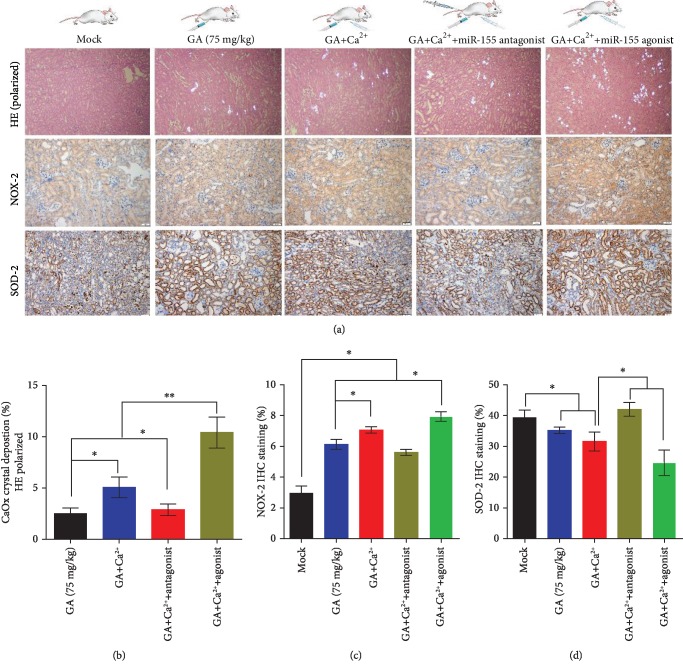
miR-155-5p promotes Ca^2+^-induced oxidative stress injury in vivo. (a) CaOx deposition in the corticomedullary junction area was measured by hematoxylin and eosin (HE) staining (polarized light microscopy), and immunohistochemical (IHC) analysis of kidney NOX2 and SOD-2 expression was performed in high-concentration Ca^2+^ combined with miR-155-5p agonist- or antagonist-injected CaOx crystal mouse model (magnification, ×200; scale bar, 50 *μ*m). (b–d) Quantification of CaOx crystal deposition level in the kidney and quantification of IHC staining of NOX2 and SOD-2 proteins after Ca^2+^, miR-155-5p agonist, antagonist, or the combination of these treatments. ^∗^*P* < 0.05 and ^∗∗^*P* < 0.01.

To elucidate the function of miR-155-5p on oxalate- and Ca^2+^-induced kidney oxidative stress injury, we intraperitoneally injected miR-155-5p agonist or antagonist in the glyoxylate-induced kidney CaOx mouse model in vivo. Using polarized light optical microphotography and immunohistochemical (IHC) staining in the kidneys, a significant increase in the CaOx crystals deposition in the high-dose oxalate and Ca^2+^ group was observed compared with that in the control group (Figures 7(a) and 8(a)). Furthermore, IHC analyses showed strong positive staining intensity for the NOX-2 protein in the high-dose oxalate- and Ca^2+^-treated mouse kidneys; moreover, miR-155-5p overexpression can further enhance its expression. However, the expression of SOD-2 protein showed weak staining in the high-dose oxalate- and Ca^2+^-treated mouse kidneys. Moreover, the expression of miR-155-5p can further attenuate SOD-2 expression (Figures 7(a)–7(d) and 8(a)–8(d)). These results are consistent with those of in vitro experiments.

## 4. Discussion

Urolithiasis is a worldwide disease, and its morbidity is increasing, but there is still a lack of understanding about the pathogenesis of nephrolithiasis in the past decades. CaOx is the main component of renal calculus, and the formation of kidney CaOx stones involves crystal nucleation, adhesive, growth, and aggregation according to the mechanism of crystal-induced renal injury [3, 4, 20]. Many other hypotheses, such as urinary supersaturation and insufficiency of lithogenic inhibitors theory and Randall's plaque theory, have tried to explain the pathogenesis of urolithiasis [9, 20].

Many studies found that an increase of ROS and NADPH oxidase in the kidney results in inflammation and injury of renal tubular cells, which may promote CaOx crystal formation [21]. Oxidative stress is the condition in which the production of ROS is greater than the protective capacity of antioxidant enzymes, such as superoxide dismutase. In recent years, the role of oxidative stress in the formation of renal calculus has received increasing attention [4, 5, 22, 23]. More and more evidence have indicated that oxidative stress-induced renal injury may be a key factor in promoting the deposition of CaOx crystal under a higher level of oxalate concentrations in the kidney, a condition seen in patients with hyperoxaluria [24, 25], which may involve inducing cell apoptosis through mitochondrial destruction with microvilli being injured and disintegrated. The consequences of these renal injuries may then induce these crystal matrices to become adhesion to the renal parenchyma [7, 20, 26]. Moreover, many studies also indicated that renal CaOx crystal deposition could be significantly decreased by antioxidant treatment in vivo [7, 27, 28]. Many other studies found that ROS is a key factor in the formation of CaOx stones by regulating a variety of signaling pathways, including the NF-*κ*B pathway [29–32]. And Liu et al. also found that the interaction between H19 and miR-216b promotes ROS and renal tubular epithelial cell injury in the process of CaOx nephrocalcinosis by HMGB1/TLR4/NF-*κ*B pathway [12]. Another study found that ROS regulate different nuclear factors such as NF-*κ*B, AP-1, and different genes c-myc and c-jun [21]. Activation of these signaling pathways results in changes in the expression of many stone-related proteins, which promote or inhibit stone formation [33, 34]. From the literature, we found that oxidative stress injury might play crucial roles in the pathogenesis of CaOx.

The major source of ROS is NADPH oxidase in the kidneys [21, 22]. NOX-derived ROS are involved in the physiological processes of the kidney, including gluconeogenesis, glucose transport, and electrolyte transport. NOX2 is the first NADPH oxidase to be described, and its only function is to produce ROS, which is probably the most typical feature of renal pathology [22, 35, 36]. Liu et al. found that H19 upregulate the expression of NOX2 but downregulate the expression of SOD-2 in CaOx mouse kidneys. Additionally, decreased IHC staining of NOX2 but strong SOD2 signals were observed in the kidney of miR-216b agonist-treated mice [12]. Qin et al. found that COM markedly increased intracellular ROS production and upregulated the expression of NOX2 and NOX4 protein in NRK-52E cells and mouse kidneys, while COM reduced cellular SOD and CAT activities [27]. These results indicated that the oxidative stress injury and activity of NADPH oxidase may play an important role in the formation of nephrolithiasis induced by oxalate and CaOx crystals. In our study, we confirmed that oxalate and COM treatment significantly increase ROS generation, LDH release, cellular MDA levels, and H_2_O_2_ concentration in HK-2 cells. Furthermore, we found that the expression of NOX2 was upregulated, while that of SOD-2 was downregulated following the treatment with oxalate and COM in vitro.

It is well known that miRNAs play a pivotal role in all kinds of processes of cell biological. Moreover, many researchers have found that miRNAs play the important role of in pathogenesis of nephrolithiasis [10]. Similarly, our results showed that the levels of miR-155-5p were remarkably increased by oxalate and calcium in HK-2 cells. Furthermore, we also found that miR-155-5p overexpression significantly increased ROS generation, LDH release, cellular MDA levels, and H_2_O_2_ concentration in HK-2 cells, while miR-155-5p inhibitor had an opposite effect. In the glyoxylate-induced kidney CaOx mouse model, our results demonstrated that miR-155-5p overexpression dramatically promoted CaOx crystals deposition in the high-dose oxalate and Ca^2+^ group compared with that in the control group. All the above results indicated that miR-155-5p knockdown dramatically reversed oxalate- and Ca^2+^-induced oxidative stress reaction and CaOx crystal formation in HK-2 cells and the kidneys by directly targeting the 3′-UTR regions of MGP.

MGP is highly expressed in the kidney, bone, and heart, and is a molecular determinant regulating extracellular matrix calcification [18, 24, 37–39]. A previous study indicated that MGP is highly expressed in calcified human atherosclerotic plaques and inhibits calcification. However, nephrolithiasis is a common ectopic calcification, similar to vascular calcification, such as the formation of calcified plaques, increased expression of calcification inhibitors, and regulating actively calcification process. MGP gene expression was detected to maintain a high level in the renal tubular epithelial cells, and crystals with multilaminated structure were formed in the injurious renal tubules with a lack of MGP expression [37, 40]. These results indicate that MGP is not only an important biomarker of atherosclerotic calcification but may also be related to renal stones formation.

In summary, we demonstrated that miR-155-5p knockdown protects the kidney against oxalate- and COM-induced oxidative stress injury via suppressing the MGP expression in vitro and in vivo. Taken together, miR-155-5p may serve as a potential target for the patients with CaOx.

## Data Availability

The data used to support the findings of this study are available from the corresponding author upon request.
